# PReS-FINAL-2119: United Kingdom survey of current management of juvenile localised scleroderma

**DOI:** 10.1186/1546-0096-11-S2-P131

**Published:** 2013-12-05

**Authors:** DP Hawley, CE Pain, EM Baildam, HE Foster

**Affiliations:** 1 Paediatric Rheumatology, NHS, Sheffield, UK; 2 Paediatric Rheumatology, NHS, Liverpool, UK; 3 Musculoskeletal Research Group, Newcastle University, Newcastle-upon-Tyne, UK

## Introduction

Juvenile localised scleroderma (JLS) is a rare condition. Researchers and clinicians seeking to improve care for children and young people (CYP) with JLS face various challenges. JLS is often difficult to assess, both at disease onset and as the condition progresses. A variety of monitoring tools (mts) have been described for JLS.

## Objectives

- To describe how mts are used and perceived by clinicians in the United Kingdom (UK).

- To describe treatments used for JLS in CYP.

## Methods

We surveyed paediatric rheumatologists (prs) and dermatologists (dms) in the UK who manage CYP with JLS, via an an e-survey distributed to the membership of two national organisations representing these clinician groups. We asked respondents for their views and experience using 15 distinct JLS mts; about transition services and treatments used.

## Results

48 clinicians (35 dms, 13 prs) from across the UK responded. At time of survey response, dms reported managing a mean 4.2 CYP with JLS; prs 17.9. 26% dms managed all CYP in collaboration (with a PR, paediatrician, adult rheumatologist or another DM) whilst 31% prs managed all CYP in collaboration (with a DM or paediatrician). 43% of dms and 91% prs reported formal transition arrangements. 9% dms and 69% prs reported involving an adult rheumatologist in transition; 8% prs involved a DM in transition.

Medical photography (100%), thermography (67%) and drawing in notes/use of pre-printed body map (both 56%) were the most regularly used mts by prs; photography (81%) drawing in notes (56%) and ultrasound (22%) were most regularly used by dms. Of 4 skin scores, the modified Rodnan skin score was the one clinicians used most frequently. However it was only used regularly by 33% prs and 3% dms. Familiarity, use (frequently or occasionally) and perceived benefit of all other skin scores was low (0-40%). Laser doppler imaging, laser doppler flowmetry and 3.0T MRI were perceived useful by larger proportions (15-70%) of clinicians than reported using them (either occasionally (0-60%) or regularly (0-11%)).

Table 1 shows reported use of specific treatments for JLS by clinician group.

**Table 1:** Shows reported use of specific treatments for JLS by clinician group

Treatment Used	Reported use by prs (% of respondents)	Reported use by dms (% of respondents)
Topical treatments Systemic steroids Ultraviolet light Methotrexate Ciclosporin Hydroxychloroquine Anti-TNF Mycophenolate mofetil Cyclophosphamide	10 90 0 100 30 40 60 90 30	100 79 68 96 32 11 4 18 0

## Conclusion

A wide range of mts are used in the UK by prs and dms managing CYP with JLS. How these tools are accessed, used and perceived varies between and within these clinician groups. There are also differences in prescribing of treatments between prs and dms. These differences will impact on the feasibility of conducting clinical trials in JLS. Further work is needed to determine accessible and validated mts for CYP with JLS. Collaboration between dms and prs is an important factor in facilitating future high quality research and standardising care in JLS.

## Disclosure of interest

None declared.

